# Co-Culture of *Monascus purpureus* and *Aspergillus niger* Isolated from Wuyi Hongqu to Enhance *Monascus* Pigments Production While Inhibiting Citrinin Production

**DOI:** 10.3390/jof11120829

**Published:** 2025-11-24

**Authors:** Qin Yu, Xi Yuan, Fusheng Chen

**Affiliations:** 1 College of Food Science and Technology, Huazhong Agricultural University, Wuhan 430070, China; yuqin@webmail.hzau.edu.cn; 2 College of Food Science and Technology, Wuhan Business University, Wuhan 430056, China

**Keywords:** co-culture, Wuyi Hongqu, *M. purpureus*, *A. niger*

## Abstract

Wuyi Hongqu (WYH), also called black-skin-red-koji, which has been utilizing as a fermentation starter for more than one thousand years in China, is a symbiotic combination of *Monascus* spp. and *Aspergillus niger* formed through long-term application and domestication. In this study, the strains of *Monascus purpureus* and *A. niger* isolated from WYH samples were used to investigate their mutual influence, especially the effects on three main secondary metabolites from *M. purpureus*, *Monascus* pigments (MPs), monacolin K (MK), and citrinin (CIT), using a double-sided Petri dish (DSPD). The results showed that co-cultivation of *M. purpureus* and *A. niger* strains was favorable to increase the MPs production while inhibiting the CIT production by *M. purpureus*, especially when *M. purpureus* strains (M_1-1_ or M_9_) were co-cultivated with certain *A. niger* strains (An_1-2_ or An_9_), respectively, and both *Monascus* strains hardly produced detectable CIT. The expression levels of CIT-related genes in *M. purpureus* M_1-1_ or M_9_ were greatly restricted when co-cultivated with *A. niger* An_1-2_ or An_9_ confirmed by RT-qPCR. This study provides important insights into the selection of WYH production strains and the effects of fungal interactions.

## 1. Introduction

Wuyi Hongqu (WYH), also called as black-skin-red-koji, is a traditional fermentation agent for the production of Chinese rice wine (Huangjiu), one of the oldest and most representative fermented alcoholic beverages in East Asia [1,2,3]. WYH has been produced and used for more than one thousand years in China [3]. WYH is a symbiotic product mainly containing *Monascus* species and *A. niger*, through long-term domestication and application, which makes it a good model for studying microbial symbiosis [4,5]. Although there has been some research about the microbial community in WYH [6,7], the microbial interaction and the resulting secondary metabolite changes remain unknown.

As one of the main fungi of WYH, *Monascus* spp. is traditionally used for making Hongqu, also called as red yeast rice or red mold rice, which has been applied as food colorants and Chinese herbal medicines for nearly two thousands of years [8,9,10], since *Monascus* spp. can produce an abundance of beneficial secondary metabolites, such as *Monascus* pigments (MPs) [10,11,12], and monacolin K (MK), also known as lovastatin, a lipid-lowering active compound [13,14]. However, some *Monascus* strains may also produce a harmful secondary metabolite, mycotoxin citrinin (CIT), affecting the food safety of Hongqu [15]. In recent years, therefore, lots of research has been conducted to increase the productions of MPs [16,17] and MK [18,19], and inhibit the CIT production [17,20].

Microbial co-culture involves two or more microorganisms growing in the same environment, which may stimulate or hinder the relative microbial growth and metabolism by triggering some regulatory mechanisms [21,22,23,24]. For example, co-culturing *M. purpureus* and *Aspergillus oryzae* can lead to the production of two novel substances by *M. purpureus*, monaspins A and B [25]. Co-culture of *Aspergillus flavus* with *Bacillus subtilis* can significantly increase the production of the anticancer drug paclitaxel by *A. flavus* [26]. Co-cultivation of *Rhodotorula mucilaginosa* and *M. purpureus* can significantly improve the yields of carotenoids of *R. mucilaginosa* and MPs of *M. purpureus* [27]. In our previous study, when *M. ruber* M7 and *Aspergillus niger* CBS 513.88 strains were jointly cultivated by double-sided Petri dish (DSPD), MPs production by *M. ruber* M7 was enhanced and two novel natural products were discovered [28].

In the current study, to investigate the interrelationship of *M. purpureus* and *A. niger* isolated from WYH, samples of traditional craftsmanship-made WYH were collected, and *M. purpureus* and *A. niger* were isolated and identified. Then, using DSPD, morphological observations were conducted, and MPs, MK, and CIT produced by *M. purpureus* were analyzed when *M. purpureus* and *A. niger* were co-cultivated. The results revealed that co-cultivation affected the growth and secondary metabolism of *M. purpureus*, promoting the production of MPs while suppressing CIT biosynthesis.

## 2. Materials and Methods

### 2.1. Samples

**Figure 1:**
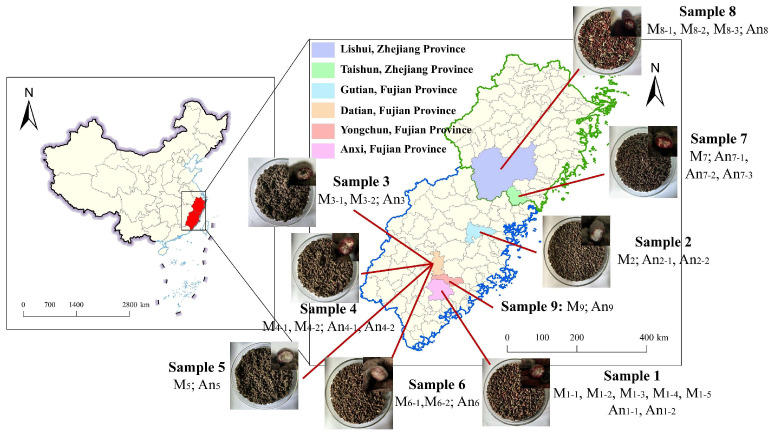
WYH samples were collected from Fujian and Zhejiang provinces of China *. *: The upper right corner of the WYH images is the cross-section of WYH grains; the M_9_ and An_9_ strains were isolated by our previous study and preserved in our laboratory; M_1-1_~M_9_: *M. purpureus* strains; An_1-1_~An_9_: *A. niger* strains.

Nine Wuyi Hongqu (WYH) samples were collected from multiple locations in Fujian and Zhejiang Provinces in China, where are the WYH main production areas (Figure 1). All samples were produced with the traditional craft method. All isolated strains were named according to the numbers of the 9 collected WYH samples. The *Monascus purpureus* strains were designated as M_1-1_ to M_9_, and the *Aspergillus niger* strains were designated as An_1-1_ to An_9_ (Figure 1).

### 2.2. Media

Potato dextrose agar (PDA): Potato (200 g/L), sugar (20 g/L), and agar power (2 g/L) [28]. PDA was used for the isolation and purification of *A. niger*, the preservation and morphological observation of isolated strains from WYH.

A total of 6% ethanol PDA: After PDA was sterilized and cooled to about 50 °C, 6% ethanol was added into PDA. It was used for the isolation and purification of *M. purpureus* [29].

Czapek yeast extract agar (CYA): NaNO_3_ (3 g/L), K_2_HPO_4_ (1 g/L), KCl (0.5 g/L), MgSO_4_·7H_2_O (0.5 g/L), FeSO_4_ (0.01 g/L), sucrose (30 g/L), yeast extracts power (1 g/L), and agar power (2 g/L).

Malt extract agar (MEA): Malt extract power (30 g/L), peptone (1 g/L), glucose (20 g/L), and agar powder (15 g/L).

A total of 25% glycerol nitrate agar (G25N): CYA with 25% glycerol. CYA, MEA, and G25N were used for morphological observation of isolated strains from WYH [28].

Rice powders broth (RB): rice power (50 g/L). RB was used for the preparation of *M. purpureus* inoculants.

Rice powders agar (RA): RB with 20g/L agar power [28], which was used for morphological observation of *M. purpureus* and *A. niger*.

All media were sterilized at 121 °C for 20 min.

### 2.3. Strain Isolation

*Monascus* spp. isolation: based on the previous method [29] and appropriately modified, 40 μL of sterile 10% lactic acid solution was added to the center of 6% ethanol PDA plate. Then, 0.5 g of WYH powder was put into the lactic acid solution. Once the lactic acid solution had been absorbed, the PDA plates were inverted and incubated at 28 °C for 3–5 d, and then single colonies were picked out and purified 3–4 times.

*Aspergillus* spp. isolation: 0.5 g of WYH powder was put into sterile water and diluted 10^−7^ to 10^−8^ times. Then, 200 μL of diluted solution was spread on the PDA plate and incubated at 28 °C for 1–2 d, and then single colonies were picked out and purified 3–4 times.

### 2.4. Colonial and Microscopic Morphologies of Strains Isolated from WYH

Preparation of strains spore suspension: The fungal strains isolated from WYH samples were cultured on PDA slants at 28 °C for 7 d, then sterile water was added into the slants and spores were scraped using an inoculating loop. After the mycelia were filtered by sterile filter paper, the spore concentration was adjusted to 1 × 10^6^ spores/mL [30].

Colonial morphological observation: 10 μL of the spore suspension was inoculated at the plate centers of PDA, MEA, CYA, G25N, and RA media, respectively. The colonial morphologies including growth rates, aerial mycelia status, and pigment production of *Monascus* spp. and *Aspergillus* spp. were observed and recorded at 28 °C on 3, 5, 7, and 9 d, respectively.

Microscopic morphological observation: 200 μL spore suspensions (10^6^ spores/mL) of *Monascus* spp. or *Aspergillus* spp. were spread on the plates of PDA, MEA, CYA, G25N, and RA media, respectively. Then, the sterilized glass slides were inserted into the media at a 45-degree angle, respectively. After incubating at 28 °C for 7 d, the slides were taken out and put under the optical microscope (BH-2, Olympus, Tokyo, Japan) to observe the microscopic morphologies such as mycelia, conidia, and cleistothecia.

### 2.5. DNA Extraction, Sequencing, and Microbial Classification

A total of 200 μL spore suspensions (10^6^ spores/mL) of *Monascus* spp. or *Aspergillus* spp. were severally spread on the PDA plates coated with sterile cellophane, and incubated for 5 d at 28 °C. Then, DNA from 20 mg mycelia was extracted using the CTAB method [29], followed by PCR amplification of the *ITS*, *LSU*, *BenA*, *CaM*, and *RPB2* genes with the corresponding primer pairs (Table S2) [31,32]. After that, the PCR products were verified by 1% agarose gel electrophoresis and sequenced by Wuhan Tianyi Huayu Gene Technology Co., Ltd. (Wuhan, China). Finally, these gene sequences were assembled in the order *ITS*-*LSU*-*BenA*-*CaM*-*RPB2* with PhyloSuite v1.2.3 [33], and multi-gene phylogenetic trees were constructed using the maximum likelihood method in IQ-TREE v2.2.0 to identify and classify the fungal strains isolated from the WYH samples.

### 2.6. Preparation for Double-Sided Petri Dish

**Figure 2:**
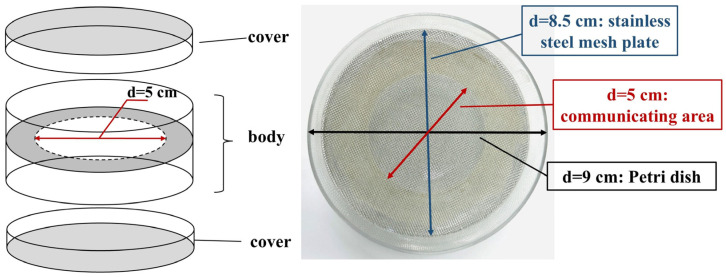
The schematic diagram of the structural composition of a double-sided Petri dish.

Based on the previous report [28], a double-sided Petri dish (DSPD) was prepared, which consists of a body (double-sided dish bottom) and two covers (Figure 2). The preparation process of DSPD is as follows: two resin Petri dish bottoms (d = 9 cm) were stuck together by glue, and a circular hole (d = 5 cm) was drilled in the center as communicating area. Then, a stainless steel mesh plate (40 mesh, d = 8.5 cm) was put into one side of the DSPD body to support the media. When DSPD was used, a sheet of sterile cellophane (d = 9 cm) was put onto the stainless steel mesh, and the media were poured into it. After the media solidified, the DSPD was reversed, and the same media were poured into another side of the DSPD body.

### 2.7. Co-Culture Methods for Fungi

Preparation of fungal inoculants: 5 mL *M. purpureus* spore suspension (10^6^ spores/mL) was inoculated in RB media and incubated at 28 °C for 7 d at 120 rpm to prepare the inoculants of *M. purpureus*. The *A. niger* spore suspension was adjusted to 1 × 10^5^ spores/mL.

Morphological observations: 10 μL *M. purpureus* inoculant and *A. niger* spore suspension were inoculated on the center of RA media on each side of DSPD, respectively, and cultivated for 7 d at 28 °C. At the same time, 10 μL of *M. purpureus* inoculant and *A. niger* spore suspension was severally inoculated on the center of RA plate of normal Petri dish as the monoculture control, respectively. The colonial morphologies of *M. purpureus* and *A. niger* of monoculture and co-culture were observed and recorded on 3, 5, and 7 d, respectively. For microscopic morphological observations, 200 μL *M. purpureus* inoculant and *A. niger* spore suspension was spread on RA plates of each side of DSPD and normal Petri dish, respectively. Then, the microscopic morphology was observed after 7 days of cultivation.

### 2.8. Detection of Secondary Metabolites

The sterile cellophane was placed on an RA plate of DSPD and the normal Petri dish, then, 200 μL of *M. purpureus* inoculant and *A. niger* spore suspension was spread on the cellophane, respectively. After incubation at 28 °C for 7 d, the mycelia and media were collected and freeze-dried (FD8-5, Golden-SIM, Miami, FL, USA), respectively. Then, the dried samples were extracted with 80% methanol solvent at 40 °C for 30 min under ultrasonication (KQ2200DE, Kunshan Ultrasonic Instrument Co., Ltd., Kunshan, China), and centrifuged (Neofuge 18R, Heal Force, Shanghai, China) at 12,000 rpm for 10 min, and the supernatants were filtered through 0.22 µm membrane (Jinteng, Tianjin, China). Thereafter, MPs, MK, and CIT were detected, respectively, according to the previously reported methods [34,35]. MPs and MK were detected using high-performance liquid chromatography (HPLC, LC-20A, Shimadzu, Kyoto, Japan) with an ODS-3 (4.6 mm × 250 mm, 5 µm) column and an injection volume of 10 μL. CIT was analyzed using ultra-performance liquid chromatography (UPLC, ACPUITY Y UPLC I-class, Waters, Milford, MA, USA) with C18 column (Waters, 2.1 × 100 mm, 1.7 μm) and an injection volume of 2 μL. Acetonitrile and 0.1% formic acid were utilized as the mobile phase with gradient elution for MPs and CIT detection [28,36]. Acetonitrile and 0.5% phosphoric acid (60:40) were applied for MK detection [37]. The contents of MPs, MK and CIT, were expressed as their peak areas (AU·min or EU·min).

### 2.9. RT-qPCR

The expression levels of MPs and CIT biosynthetic genes in *M. purpureus* under both monoculture and co-culture were quantified by RT-qPCR analyses. Total RNA was extracted from mycelia cultured at 28 °C on RA for 5 d using the TransZol Up Plus RNA Kit (TransGen Biotech, Beijing, China). RNA was converted to cDNA using the HiScript II 1st Strand cDNA Synthesis Kit (Vazyme, Nanjing, China), with the beta-actin gene as an internal control (Table S5). RT-qPCR detection was performed using the AceQ qPCR SYBR Green Master Mix (Vazyme, Nanjing, China) [36]. The relative expression levels of genes relative to MPs and CIT biosynthesis were calculated following the previously described formula [38].

## 3. Results

### 3.1. Isolation and Classification of Fungal Strains from WYH Samples

Nine Wuyi Hongqu (WYH) samples (Figure 1) made by with the traditional handmade craft were collected from Fujian and Zhejiang Provinces in China, which are the main production areas of WYH [3]. Except Sample 8, the grains surface features of WYH samples exhibited uniform black–red (Sample 1), gray–black (Samples 2, 4, and 6), and deep black colors (Samples 3, 5, and 7), and the cross-section properties of WYH grains were black coat, red wall, and white heart (Figure 1).

**Figure 3:**
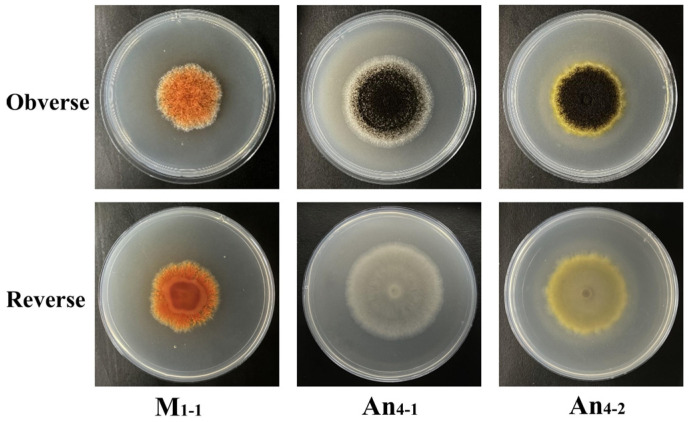
Colonial morphologies of typical putative *Monascus* and *Aspergillus* strains isolated from WYH. M_1-1_ was typical putative *Monascus* strain, An_4-1_ was the non-pigment-producing *Aspergillus* strain, and A_4-2_ was the pigment-producing *Aspergillus* strain. The putative *Monascus* and *Aspergillus* strains in the above colonies were cultured at 28 °C for 9 d and 5 d, respectively.

A total of 32 fungal strains were isolated from the WYH samples. Among them, 18 strains exhibited the characteristic morphologies of *Monascus* spp., while 14 strains exhibited morphologies consistent with *Aspergillus niger*. The colonial and microscopic morphologies of the fungal strains were observed and compared on PDA, CYA, MEA, G25N, and RA media, respectively, and the colonial and microscopic photos of these strains are shown in Figure S1, and their characters are described in Table S1. The colonial morphologies of the putative *Monascus* strains in above-mentioned different media were same as those reported in previous research [28,39], and putative *Aspergillus* strains were simply divided into pigment-producing *Aspergillus* strains and non-pigment-producing *Aspergillus* strains based on the colors of the margin and reverse sides of colonies (Figure S1). The morphologies of three typical strains of *Monascus* and *A. niger* are shown in Figure 3. Microscopic examination revealed slender, septate vegetative mycelia for putative *Monascus* strains, with abundant granules exhibiting orange, yellow and red colors, and cleistothecia and conidia formation was significantly influenced by media. For example, no cleistothecium was observed on CYA or G25N media (Figure S2). For putative *Aspergillus* strains, their conidiophores were mainly spherical and black, but some of them were orange–yellow and reddish–brown (Figure S2, Table S1).

**Figure 4:**
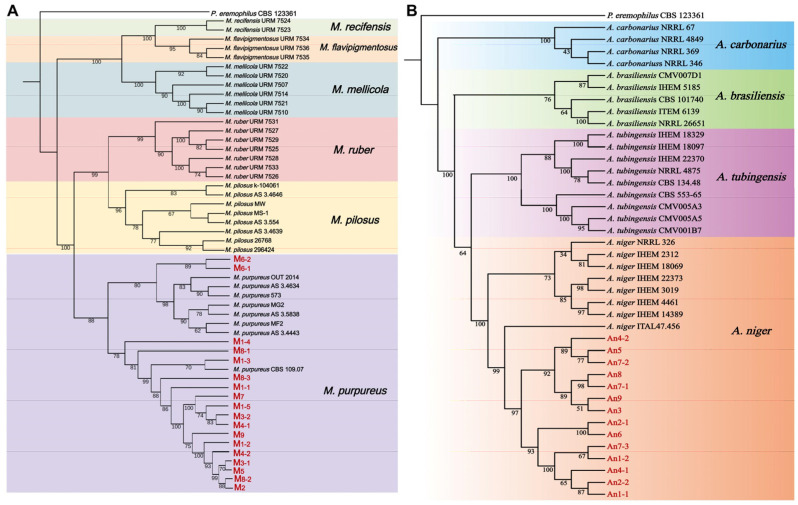
Phylogenetic trees based on multiple genes of the putative *Monascus* and *Aspergillus* strains. (**A**): Phylogenetic tree of *Monascus* strains based on *ITS*-*LSU*-*BenA*-*CaM*-*RPB2*; (**B**): phylogenetic tree of *Aspergillus* strains based on *ITS*-*BenA*-*CaM*-*RPB2*.

In order to classify and identify the isolated fungal strains, the *ITS*, *LSU*, *BenA*, *CaM*, and *RPB2* genes from the isolated fungal strains from WYH samples were amplified and sequenced (Table S3), and multi-gene joint phylogenetic trees (Figure 4) were constructed to classify these strains through combining *ITS*-*LSU*-*BenA*-*CaM*-*RPB2* gene sequences based on the DNA sequences from reference strains (Table S4) and the tested fungal strains. Due to the lack of *LSU* sequences from reference strains of *Aspergillus* spp. [31], the isolated putative *Aspergillus* strains were identified by *ITS*-*BenA*-*CaM*-*RPB2* gene sequences. The results showed that all putative *Monascus* and *Aspergillus* strains were identified as *M. purpureus* and *A. niger*, respectively.

### 3.2. Morphological Properties and Biomass in Co-Culture of M. purpureus and A. niger

Based on the morphological characteristics of *M. purpureus* and *A. niger* (Table S1, Figure S1), six strains of *M. purpureus* and seven strains of *A. niger* from different WYH samples were selected. To study the effects of fungal interactions on growth and metabolism of *M. purpureus*, 14 groups of fungal co-culture experiments were conducted on RA media using DSPD. The combinations of *M. purpureus* and *A. niger* strains are shown in Table 1.

**Figure 5:**
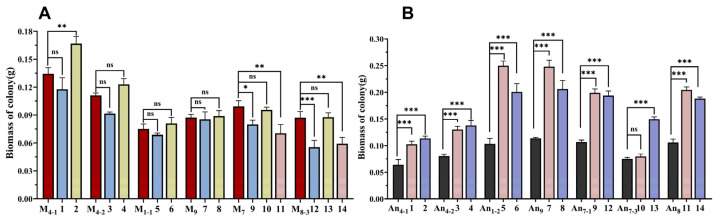
Biomasses of *M. purpureus* and *A. niger* after 7 d of co-cultivation on RA media. (**A**): Biomass of *M. purpureus*; (**B**): biomass of *A. niger*. The data are represented as the mean ± standard error (n = 3). * *p* < 0.05, ** *p* < 0.01, *** *p* < 0.005, ns: not significant compared to monoculture.

*M. purpureus* and *A. niger* were co-cultured on a double-sided Petri dish (DSPD), and their colonial and microscopic morphologies are shown in Figures S4 and S5, and their morphological characters are described in Table 2, and their biomasses are revealed in Figure 5. Compared with monoculture, the colonies of *M. purpureus* were smaller and orange in the co-culture (red in monoculture) and the numbers of conidia and cleistothecia were changed, while the colonies of *A. niger* after co-culture were larger than monoculture and there was no significant difference in micro-morphologies. Specially, when *M. purpureus* was co-cultured with pigment-producing *A. niger*, they possessed larger colonies than those with non-pigment-producing *A. niger* (Figure S5). After co-cultivation, the biomasses of most *M. purpureus* decreased slightly, but not significantly, while the biomasses of *A. niger* increased significantly (Figure 5). Among them, when *M. purpureus* was co-cultured with pigment-producing *A. niger* (An_4-1_ and An_7-3_), the biomasses of *M. purpureus* increased, especially in *M. purpureus* strains (M_4-1_ and M_8-3_) with weaker pigment-production capacities.

### 3.3. Co-Cultivation Effects on M. purpureus Secondary Metabolites

**Figure 6:**
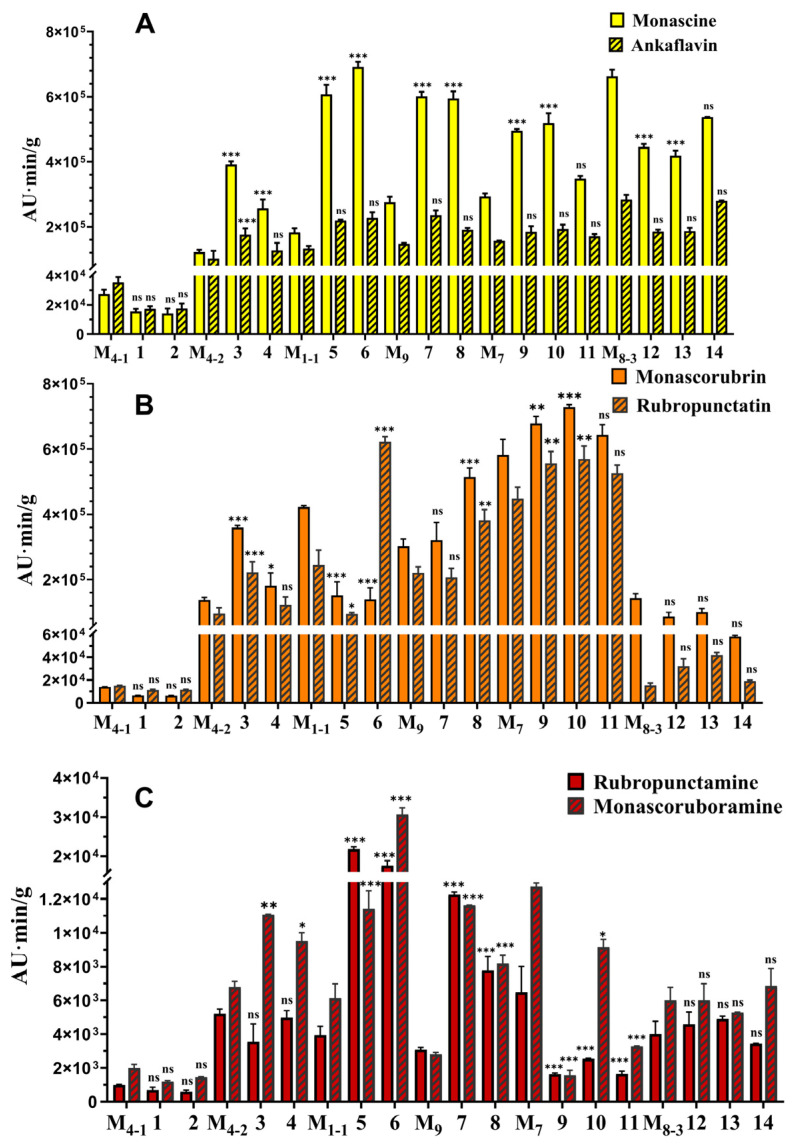
MPs produced by *M. purpureus* co-cultured with *A. niger* on RA media for 7 d. (**A**): *M. purpureus* YPs; (**B**): *M. purpureus* OPs; (**C**): *M. purpureus* RPs. The data were represented as the mean ± standard error (n = 3). * *p* < 0.05, ** *p* < 0.01, *** *p* < 0.005, ns: not significant compared to monoculture. AU: Absorbance Unit, AU·min: peak area.

**Figure 7:**
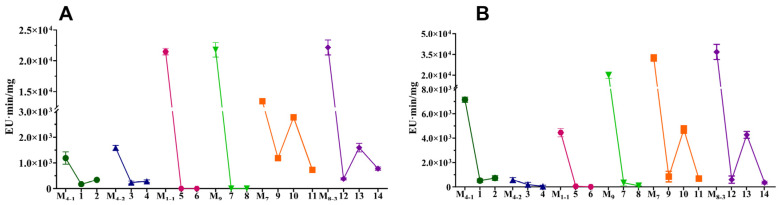
CIT produced by *M. purpureus* co-cultured with *A. niger* on RA media for 7 d. (**A**): Intracellular CIT; (**B**): extracellular CIT. EU: Electrical Unit, EU·min: peak area.

To further investigate the effects of *A. niger* on the secondary metabolites (SMs) of *M. purpureus*, its main SMs including *Monascus* pigments (MPs), monacolin K (MK), and citrinin (CIT) [28] were analyzed. The results (Figure 6) showed that after co-cultivation with *A. niger* for 7 d, all *M. purpureus* strains in monoculture and co-culture did not produce MK (the results not revealed), and the main six classic MPs including yellow pigments (YPs, Monascin and Ankaflavin), orange pigments (OPs, Rubropunctatin and Monascorubrin) and red pigments (RPs, Rubropunctamine and Monascorubramine) [12,40] were increased to varying degrees, except M_4-1_ and M_8-3_ (Figure 6). CIT detection revealed that after co-cultivation of *M. purpureus* and *A. niger*, both intracellular and extracellular CIT contents were significantly reduced, decreasing to 0–28.57% and 0–45.71%, compared to monoculture, respectively (Figure 7). Among them, when *M. purpureus* M_1-1_ and M_9_ were co-cultured with *A. niger* An_1-2_ and An_9_, respectively, they produced undetectable citrinin. Additionally, when co-cultured with non-pigment-producing *A. niger*, the pigment yields of *M. purpureus* significantly increased and the contents of CIT significantly decreased, but when co-cultured with pigment-producing *A. niger*, these changes were inhibited to a certain extent.

### 3.4. RT-qPCR for Analyzing Expression Levels of Genes Relative MPs and CIT Production

**Figure 8:**
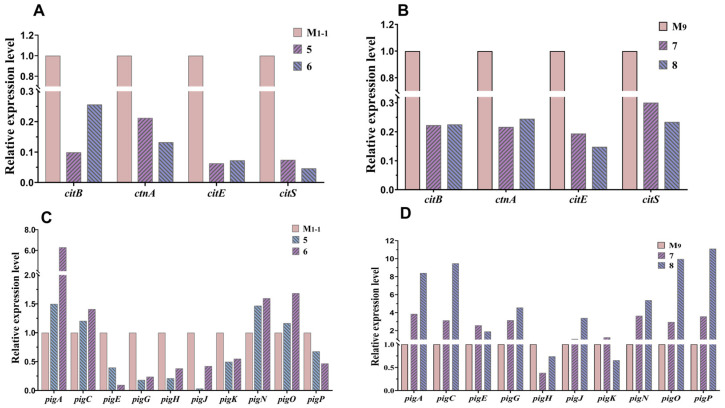
Expression levels of CIT and MPs biosynthetic-related genes of *M. purpureus* M_1-1_ and M_9_, under monoculture and co-culture with *A. niger* An_1-2_ and An_9_. (**A**,**B**): CIT gene expression levels; (**C**,**D**): MPs gene expression levels. Except for the genes in the figure, other genes were poorly expressed or unexpressed and are not shown in the results. Here, 5: M_1-1_ and An_1-2_, 6: M_1-1_ and An_9_, 7: M_9_ and An_1-2_, and 8: M_9_ and An_9_.

After co-culturing *M. purpureus* M_1-1_ and M_9_ with *A. niger*, respectively, they produced undetectable intracellular and extracellular CIT and significantly higher yields of MPs (Figure 6 and Figure 7). Therefore, the expression levels of key genes [12,40,41] in the CIT and MPs biosynthetic gene clusters of *M. purpureus* M_1-1_ and M_9_ in mono- and co-culture were analyzed and compared. As determined by RT-qPCR, the expression levels of key genes relative to CIT in strains M_1-1_ and M_9_ were significantly reduced after co-culture (Figure 8A,B). Meanwhile, the expression levels of MPs after co-culture of M_1-1_ and M_9_ increased and decreased to varying degrees (Figure 8C,D). The gene expression levels of M_1-1_ and M_9_ after co-culture were consistent with the previous results of CIT and MPs contents (Figure 6 and Figure 7).

## 4. Discussion

WYH is a fungal co-cultivation fermentation product mainly including *Monascus* spp. and *Aspergillus niger*, and is used in the production of yellow wine and *Monascus* vinegar in China [3,28]. Up to now, the co-cultivation of *Monascus* spp. and *A. niger* from WYH has had little research. A double-sided Petri dish (DSPD) provides an interface for communication between *M. purpureus* and *A. niger*, facilitating the separation of mycelia for metabolite analyses, and can also simulate the exchange state between these two fungi during WYH fermentation [28]. When co-cultured using DSPD, *A. niger* exhibited a significant effect on the growths (Figures S4 and S5) and metabolite productions (Figure 6 and Figure 7) of *M. purpureus*. However, co-cultivation using DSPD represents an indirect approach in which metabolites were exchanged through the media, whereas certain interactions may only occur during direct physical contact [42]. Therefore, various direct co-culture strategies, including the simulation of WYH fermentation on rice grains, are being investigated to further elucidate the interaction mechanisms between *M. purpureus* and *A. niger*.

As an important secondary metabolite of *Monascus* spp., Monacolin K (MK) was not detected in any of the examined *M. purpureus* strains examined. Previous genomic analyses have suggested that *Monascus* strains are unlikely to simultaneously produce MPs, MK, and CIT, as the complete biosynthetic gene clusters responsible for these metabolites rarely coexist within the same genome [43]. Furthermore, MK biosynthesis appeared to be species-dependent: *M. ruber* and *M. pilosus* possessed conserved and complete MK biosynthetic gene clusters, whereas *M. purpureus* usually lacked a complete MK biosynthetic gene cluster [44,45,46,47]. These findings are consistent with the results of the present study.

Previously, co-cultivation of *M. ruber* M7 with *A. niger* CBS 513.88 led to the discovery of two novel compounds [28]. In the present study, two similar compounds were also detected in the products of *M. purpureus* M_1-1_ co-cultured with *A. niger*, which are currently being isolated and characterized. These findings suggest that co-cultivation of *M. purpureus* with *A. niger* not only influences fungal growths and metabolic activities, but may also activate silent gene clusters to yield additional secondary metabolites. In the future, expanding fungal co-culture approaches may provide opportunities for the discovery of further novel secondary metabolites (SMs) [48]. It is noteworthy that analytical techniques such as high-performance liquid chromatography (HPLC) and mass spectrometry (MS) are commonly employed for metabolite detection in co-culture systems [23]. However, the concentrations of novel SMs produced under co-cultivation conditions are often relatively low, which may limit their detection by conventional instrumental methods. Therefore, enhancing SMs extraction efficiency and applying more sensitive and precise analytical platforms are recommended for subsequent investigations.

In this study, the growths and pigment-producing capacities of *M. purpureus* were found to exert a substantial influence on the outcomes of co-cultivation. For instance, strains M_4-1_ and M_8-3_, which exhibited relatively weak pigment production, showed a decline in pigment yields after co-cultivation. By contrast, strains with strong growth potentials, such as M_1-1_ and M_9_, displayed increased pigment production and decreased CIT (Figure 6 and Figure 7). These results highlight the critical importance of strain selection in WYH production. Specifically, the use of *M. purpureus* strains with strong pigment-producing abilities, in combination with non-pigmenting *A. niger* strains, is recommended to ensure high-quality and safe WYH products. In addition, MK, a lipid-lowering secondary metabolite produced by *Monascus* spp., represents another valuable target for strain selection. Thus, co-cultivation of *Monascus* strains with high MK biosynthetic potential and *A. niger* could provide a promising strategy for developing functional WYH in the future. Nevertheless, our previous experiments revealed that co-cultivation of the high MK-producing *Monascus* strain MS-1 with *A. niger* CBS 513.88 or CBS 113.46 failed to yield detectable levels of MK [28]. These observations suggest that further research is necessary to determine whether high MK-producing *Monascus* strains are suitable for WYH fermentation.

In addition, owing to the substantial variability among strains and the lack of a clear classification system for *A. niger* SMs [49], the impact of co-cultivation on *A. niger* SMs was not addressed in this study. Nevertheless, differences were observed in the effects of pigment-producing and non-pigment-producing *A. niger* strains on the SMs of *M. purpureus* (Figure 6). In particular, pigment-producing strains of *A. niger* (An_4-2_ and An_7-3_) appeared to suppress the increase in MPs and attenuate the decrease in CIT. Therefore, the principal SMs produced by pigment-producing and non-pigmenting *A. niger* strains are currently being isolated and identified to provide deeper insights into the mechanisms through which signaling molecules mediate interspecies interactions.

## 5. Conclusions

This study revealed the interaction between *Monascus purpureus* and *Aspergillus niger* through co-cultures of *M. purpureus* and *A. niger* isolated from WYH, and the effects of *A. niger* on the growths and metabolites of *M. purpureus*. Morphological results showed that *M. purpureus* and *A. niger* have significantly changed after co-cultivation. Metabolite analysis demonstrated that *A. niger* could promote the production of MPs and inhibits the production of CIT. The results of this study provide useful guidance for the selection of strains for production of WYH and serves as a good model for further research to enhance MPs production while reducing CIT levels.

## Figures and Tables

**Table 1:** The co-culture experimental combination for *M. purpureus* and *A. niger*.

No. of Co-Culture	Strain Names	Strain Sources	Pigment-Production Abilities	Growth Abilities
1	M_4-1_ & An_4-1_	Sample 4	M_4-1_(+)	M_4-1_(+)
2	M_4-1_ & An_4-2_		M_4-2_(++)	M_4-2_(++)
3	M_4-2_ & An_4-1_		An_4-1_(−)	An_4-1_(+++)
4	M_4-1_ & An_4-2_		An_4-2_(+)	An_4-2_(++)
5	M_1-1_ & An_1-2_	Sample 1 and Sample 9	M_1-1_(++)	M_1-1_(++)
6	M_1-1_ & An_9_		M_9_(++)	M_9_(++)
7	M_9_ & An_1-2_		An_1-2_(−)	An_1-2_(+++)
8	M_9_ & An_9_		An_9_(−)	An_9_(+++)
9	M_7_ & An_7-1_	Sample 7 and Sample 8	M_7_(++) M_8-3_(+) An_7-1_(−) An_7-3_(+) An_8_(−)	M_7_(++) M_8-3_(+) An_7-1_(+++) An_7-3_(++) An_8_(+++)
10	M_7_ & An_7-3_			
11	M_7_ & An_8_			
12	M_8-3_ & An_7-1_			
13	M_8-3_ & An_7-3_			
14	M_8-3_ & An_8_			

+: indicates pigment production, and the number of ‘+’ symbols represents the relative strength of pigment-producing and growth abilities; −: indicates no pigment production.

**Table 2:** The colonial and microscopic morphologies of *M. purpureus* and *A. niger* on co-culture using DSPD on RA media.

Strain Types	Culture Types	Colonial Sizes	Colonies Colors	Aerial Hyphae	Pigment Granules Shapes	Cleistothecia Number	Conidia Number
*M. purpureus*	Monoculture	++	red	+	Combs. 1, 2, 10, 11: block-like to needle-like; Comb. 5: needle -like to block -like	Combs. 2, 12, 13: increased.	Combs. 7, 8: increased; Com3,4: decreased.
	Co-culture	+	orange or yellow	++			
*A. niger*	Monoculture	++	black	/	/	/	/
	Co-culture	+++	black	/	/	/	/

+: the number indicates colonies size or aerial hyphae relative change of monoculture and co-culture; /: no relevant morphological characteristics or no significant change.

## Data Availability

The original contributions presented in this study are included in the article/Supplementary Materials. Further inquiries can be directed to the corresponding authors.

## Supplementary Materials

The following supporting information can be downloaded at: https://www.mdpi.com/article/10.3390/jof11120829/s1, Figure S1: Colonial morphologies of strains isolated from WYH on different media; Figure S2: Microscopic morphologies of *Monascus* spp. and *Aspergillus* spp. isolated from WYH; Figure S3: Agarose electrophoresis of PCR amplification products; Figure S4: Colonial morphologies of *M. purpureus* and *A. niger* co-cultured using DSPD on RA media at 28 °C; Figure S5: Microscopic morphologies of *M. purpureus* and *A. niger* co-cultured using DSPD on RA media at 28 °C; Figure S6: HPLC chromatography diagram of MPs; Figure S7: HPLC chromatography diagram of *Monascus* red pigments; Figure S8: UPLC chromatography diagram of intracellular CIT; Figure S9: UPLC chromatography diagram of extracellular CIT; Figure S10: Total RNA of 6 samples for RNA in 1% agarose gel electrophoresis; Table S1: The colonial and microscopic morphologies of putative *Monascus* and *Aspergillus* strains on different media; Table S2: Primers for gene amplification and fragment length; Table S3: GenBank accession No. of *M. purpureus* and *A. niger* strains; Table S4: Strains and sequences used in molecular study; Table S5: Primers for RT-qPCR of *Monascus* MPs and CIT biosynthetic genes.
