# Supplementary material for: Co-Culture of Monascus purpureus and Aspergillus niger Isolated from Wuyi Hongqu to Enhance Monascus Pigments Production While Inhibiting Citrinin Production
Source: J Fungi (Basel). 2025 Nov 24;11(12):829. doi: 10.3390/jof11120829 (PMC12733441; doi:10.3390/jof11120829)
Supplement: Supplementary file 1 [file jof-11-00829-s001.zip › jof-3961051-supplementary.pdf]

Co-culture of *Monascus purpureus* and *Aspergillus niger* isolated from Wuyi Hongqu to enhance *Monascus* pigments production while inhibit citrinin production

Qin Yu [1], Xi Yuan [2,*] and Fusheng Chen [1,*]

[1] College of Food Science and Technology, Huazhong Agricultural University, Wuhan 430070, China; yuqin@webmail.hzau.edu.cn
[2] College of Food Science and Technology, Wuhan Business University, Wuhan 430056, China; yuanxiraiden@163.com
* Correspondence: chenfs@mail.hzau.edu.cn; Tel.: +86-13986273471

1. Colonial and microscopic morphologies of isolated strains 2
2. PCR electrophoresis validation and DNA sequence 5
2.1 DNA sequencing related primers and PCR amplification procedures 5
2.2 Electrophoresis of PCR amplification products 6
2.3 DNA sequence information 7
2.4 Sequence of reference strains 8
3. Morphological observation 10
3.1 Colonial morphologies results 10
3.2 Microscopic morphologies results 11
4. High performance liquid chromatography 12
4.1 Liquid chromatography of MPs 12
4.2 Liquid chromatography of CIT 14
4.2.1 Intracellular CIT 14
4.2.2 Extracellular CIT 15
5. RT-qPCR 16
5.1 Electrophoresis of total RNA 16
5.2 RT-qPCR related primers 16

1. Colonial and microscopic morphologies of isolated strains

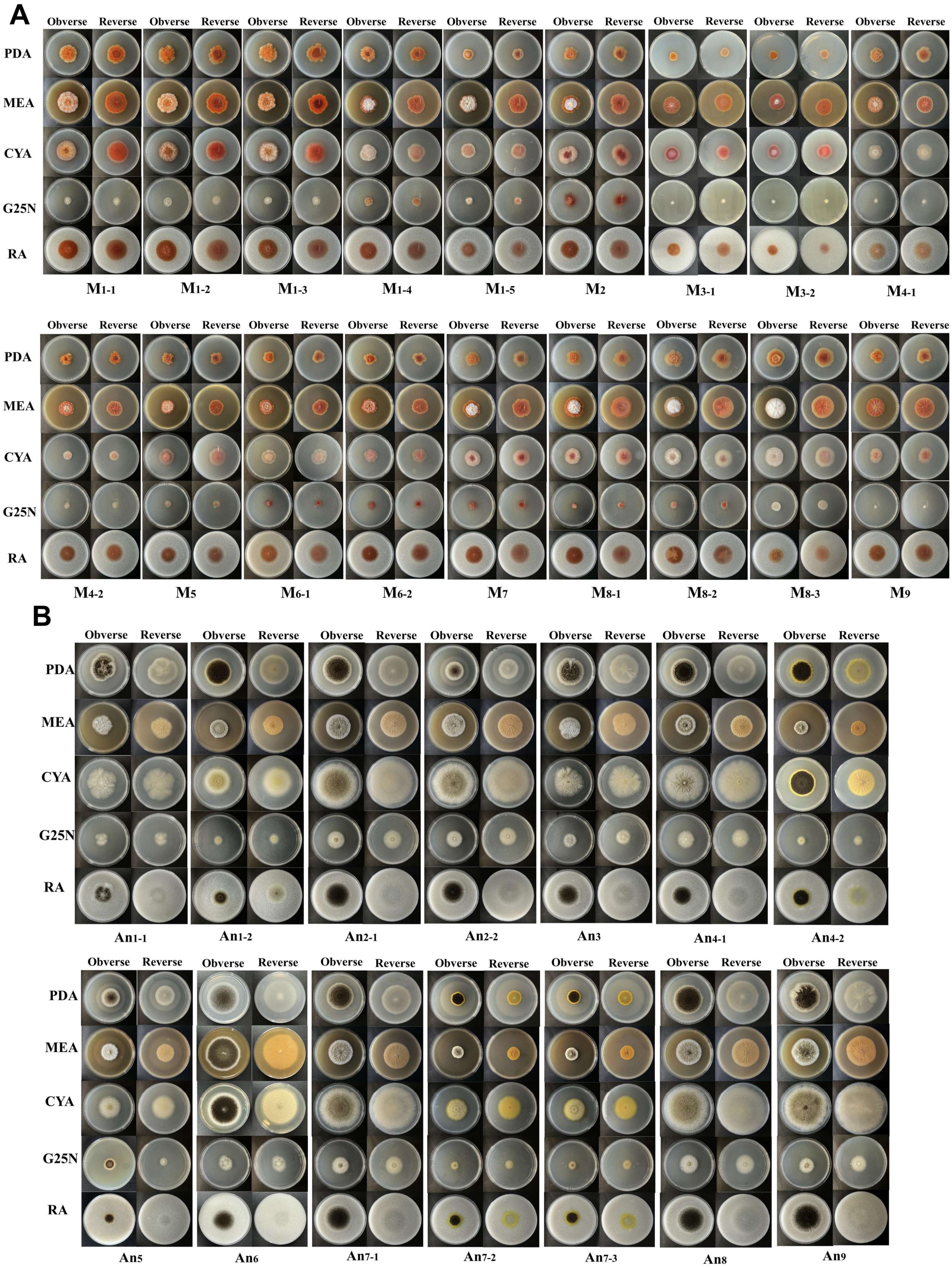

Figure S1 Colonial morphologies of strains isolated from WYH on different media

A: Colonial morphologies of *Monascus* spp. after culturing at 28℃ for 9 d; B: Colonial morphologies of *Aspergillus* spp. after culturing at 28℃ for 5 d. Obverse: Colonial morphologies on the front side of the media; Reverse: Colonial morphologies on the back side of the media. PDA, MEA, CYA, G25N and RA: five media used for colonial morphologies observation of isolated strains.

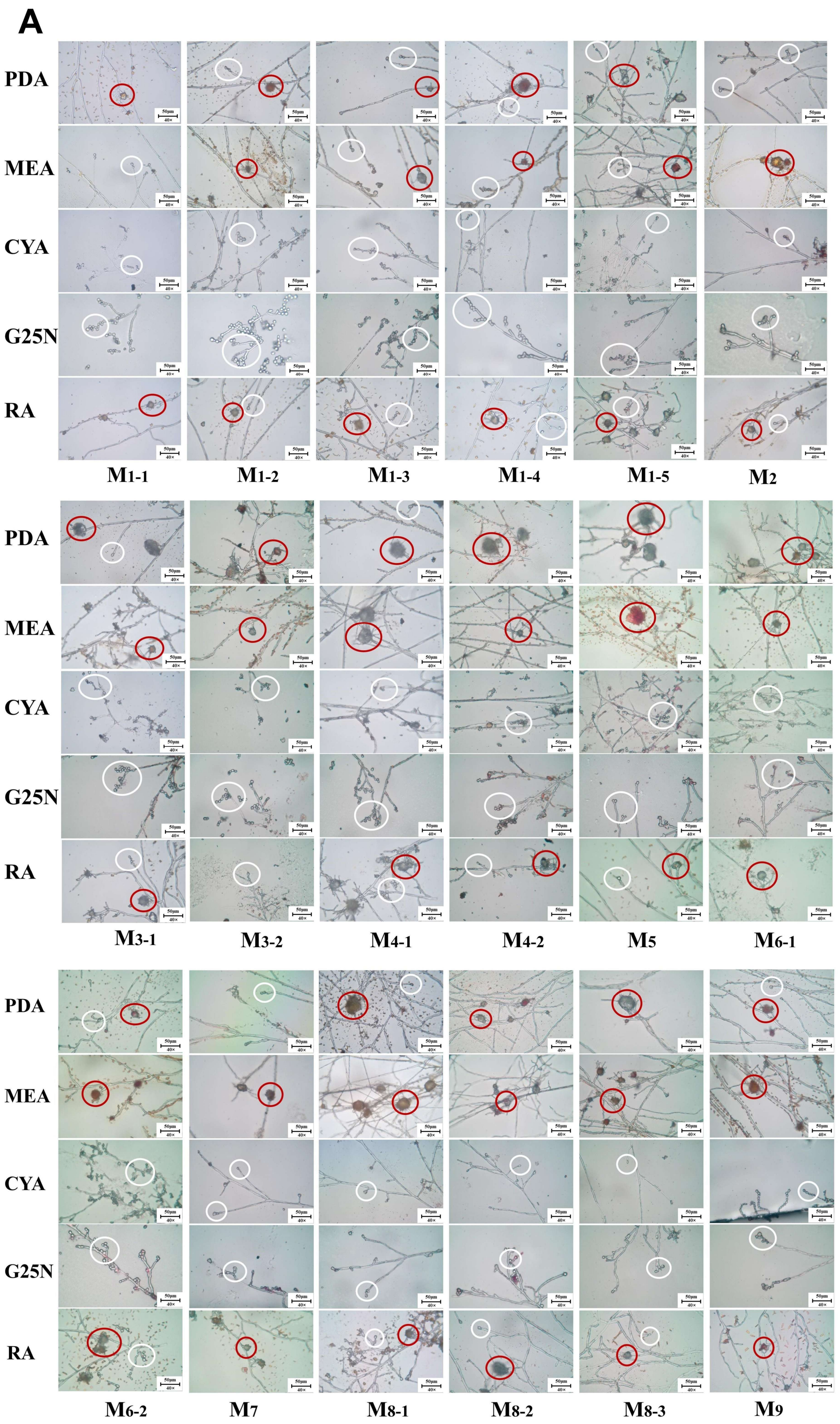
A
PDA
MEA
CYA
G25N
RA
M1-1
M1-2
M1-3
M1-4
M1-5
M2
M3-1
M3-2
M4-1
M4-2
M5
M6-1
M6-2
M7
M8-1
M8-2
M8-3
M9

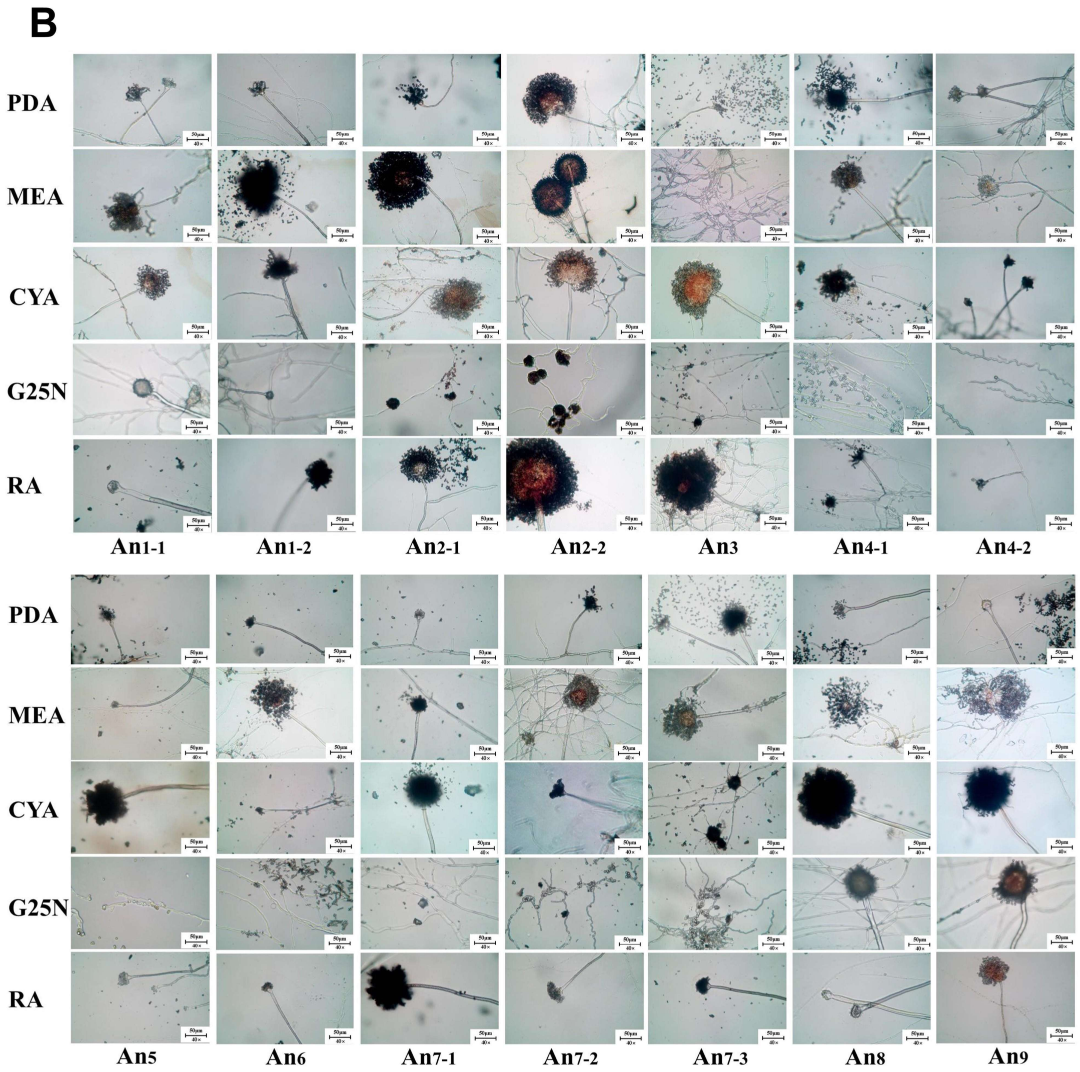

Figure S2 Microscopic morphologies of *Monascus* spp. and *Aspergillus* spp. isolated from WYH

A: Microscopic morphologies of *Monascus* spp. after culturing at 28°C for 7 d; B: Microscopic morphologies of *Aspergillus* spp. after culturing at 28°C for 7 d. PDA, MEA, CYA, G25N and RA: five media used for colonial morphologies observation of isolated strains.

Table S1 The colonial and microscopic morphologies of putative *Monascus* and *Aspergillus* strains on different media

Strain types	Media	Colonial and microscopic morphologies
***Monascus* spp.**	PDA	The front and back sides of the colonies of the tested strains were orange, with abundant aerial hyphae, and the colonial sized of Strain $M_{1\text{-}1}$, $M_{1\text{-}2}$, $M_{1\text{-}3}$, $M_{8\text{-}3}$ were larger than those of others. All strains could produce the conidia and cleistothecia.
	MEA	All strains' colonies showed radial stripes, and some colonies had an orange front side while the reverse side appeared orange-red or red; the remaining colonies had white centers with orange edges, only the colony of Strain $M_{8\text{-}3}$ was entirely white, and only Strain M_9 with few aerial hyphae. All strains could produce conidia and cleistothecia.
	CYA	Most strains had pink colonies on the front and back sides, while Strain $M_{4\text{-}1}$, $M_{8\text{-}2}$, and $M_{8\text{-}3}$ were white on both sides. The colonial sizes of

		Strain M_{1-1}, M_{1-2}, M_{1-3}, M_2, and M_{8-3} were larger than those of other strains. In terms of microscopic morphologies, only conidia could be observed.
	G25N	All tested strains' colonies were pink and white, and compared with other media, the colonies on G25N were the smallest. In terms of microscopic morphologies, only conidia could be found.
	RA	Most of tested strains' colonies were red, except that the colonies of Strains M_{4-1} and M_{8-3} were orange, and Strains M_{8-3} had abundant aerial hyphae. All strains had conidia and cleistothecia.
***Aspergillus* spp.**	PDA	The colonies were mainly black with white edges. And Strains An_{4-2}, An_{7-2}, and An_{7-3} possessed yellow colonial edges with smaller colonies, and yellow pigment deposition on the reverse sides. All strains possessed conidiophores and conidia.
	MEA	The colonies were radial with colors ranging from gray, black to white. And the colonial sides of Strain An_{1-2}, An_{4-2}, An_5, An_{7-2}, and An_{7-3} were smaller than those of other strains. All strains showed conidial heads of various shapes and abundant black conidia.
	CYA	The colonies exhibited rich colors including white, gray, yellow and black. And the strains grew the fastest with larger colonies comparing with other media. In terms of microscopic morphologies, all tested strains possessed the large conidial heads and abundant conidia with various colors such as black, red and brown.
	G25N	The colonies were white, black and yellow with the smallest sizes comparing with other media. In terms of microscopic morphologies, only a few strains could possess complete and small conidiophores, and the number of conidia was significantly reduced compared to those in other media.
	RA	The colonies were mainly black. The morphologies of the three pigment-producing strains (An_{4-2}, An_{7-2}, and An_{7-3}) were similar to those on PDA. And the colonial sizes of Strain An_{1-2}, An_{4-2}, An_5, An_{7-2}, and An_{7-3} were smaller than those of other strains. All strains had conidiophores and abundant conidia.

2. PCR electrophoresis validation and DNA sequence

2.1 DNA sequencing related primers and PCR amplification procedures

Table S2 Primers for gene amplification and fragment length

Gene	Primers	Primer sequences (5′-3′)	Fragment length
ITS	ITS1	TCCGTAGGTGAACCTGCGG	~550bp
	ITS4	TCCTCCGCTTATTGATATGC	
LSU	LR0R	ACCCGCTGAACTTAAGC	~900bp
	LR5	TCCTGAGGGAAACTTCG	
BenA	Bt2a	GGTAACCAAATCGGTGCTGCTTTC	~520bp
	Bt2b	ACCCTCAGTGTAGTGACCCTTGGC	
CaM	Cmd5	CCGAGTACAAGGA**R**GCCTTC	~520bp
	Cmd6	CCGAT**R**GAGGTCAT**R**ACGTGG	
RPB2	5Feur	GA**Y**GA**Y**CG**K**GA**Y**CA**Y**TTCGG	~1200bp
	7CReur	CCCAT**R**GC**Y**TG**Y**TT**R**CCCAT	

The degenerate base R represents either A or G; The degenerate base Y represents either C or T; The degenerate base K represents either G or T.

PCR amplification using a 25 μL reaction system:

10×PCR buffer	2.5 μL
dNTPs	2.0 μL
Primers	1.0 μL
DNA	0.5 μL
Easy Taq DNA polymerase	0.2 μL
ddH_2O	17.8 μL

The PCR amplification procedures were: pre-denaturation at 94°C for 5 min, denaturation at 94°C for 45 s, annealing at 52°C for 45 s, extension at 72°C for 60 s, cycling for 35 times, and finally extending at 72°C for 10 min and storage at 4°C.

2.2 Electrophoresis of PCR amplification products

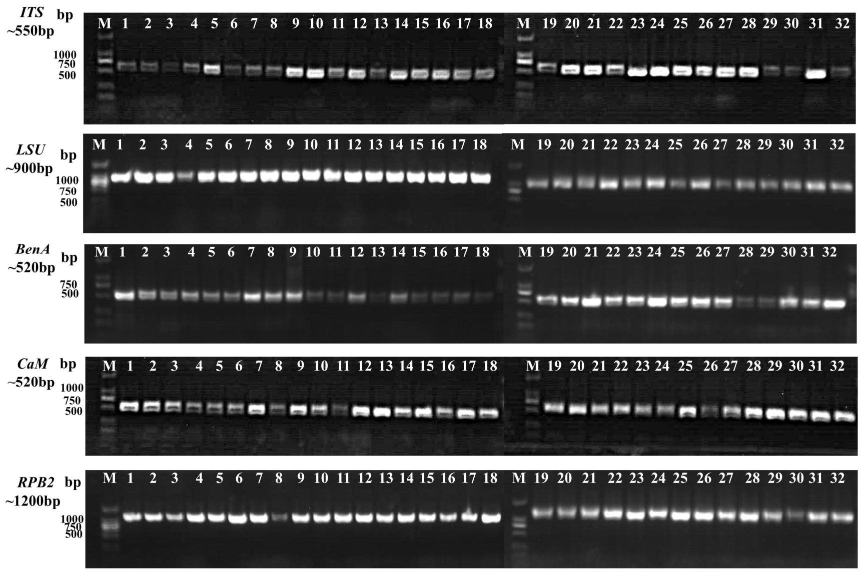

Figure S3 Agarose electrophoresis of PCR amplification products

M: *Trans* 2K DNA Maker; Lane 1~18: *Monascus* spp. $M_{1\text{-}1}$~M_9; Lane 19~32: *Aspergillus* spp. $An_{1\text{-}1}$~An_9

2.3 DNA sequence information

Table S3 GenBank accession No. of *M. purpureus* and *A. niger* strains

Species	Strains No.	GenBank accession No.				
		ITS	***BenA***	***LSU***	***CaM***	***RPB2***
Monascus purpureus	M_{1-1}	PX557985	PX576677	PX567922	PX576655	PX578627
	M_{1-2}	PX557970	PX576678	PX567923	PX576656	PX578628
	M_{1-3}	PX557971	PX576679	PX567924	PX576657	PX578629
	M_{1-4}	-	PX576680	PX567925	PX576658	PX578630
	M_{1-5}	PX557972	PX576681	PX567926	PX576659	PX578631
	M_{2}	PX557984	PX576675	PX567938	PX576653	PX578643
	M_{3-1}	PX557973	PX576682	PX567927	PX576660	PX578632
	M_{3-2}	PX557974	PX576683	PX567928	PX576661	PX578633
	M_{4-1}	PX557975	PX576684	PX567929	PX576662	PX578634
	M_{4-2}	PX557976	PX576685	PX567930	PX576663	PX578635
	M_{5}	PX557977	PX576686	PX567931	PX576664	PX578636
	M_{6-1}	PX557978	PX576687	PX567932	PX576665	PX578637
	M_{6-2}	PX557979	PX576688	PX567933	PX576666	PX578638
	M_{7}	PX557983	PX576674	PX567937	PX576670	PX578642
	M_{8-1}	PX557980	PX576671	PX567934	PX576667	PX578639
	M_{8-2}	PX557981	PX576672	PX567935	PX576668	PX578640
	M_{8-3}	PX557982	PX576673	PX567936	PX576669	PX578641
	M_{9}	PX557969	PX576676	PX567921	PX576654	PX578626
Aspergillus niger	An_{1-1}	PX557993	PX576698	PX567944	PX576704	PX578654
	An_{1-2}	PX557994	PX576699	PX567945	PX576705	PX578655
	An_{2-1}	PX558002	PX576693	PX567952	PX576712	PX578649
	An_{2-2}	PX558003	PX576694	PX567953	PX576713	PX578650
	An_{3}	PX558004	PX576695	PX567943	PX576714	PX578651
	An_{4-1}	PX557995	PX576700	PX567946	PX576706	PX578656
	An_{4-2}	PX557996	-	PX567947	PX576707	PX578657
	An_{5}	PX558005	PX576696	PX567954	PX576715	PX578652
	An_{6}	PX558001	PX576692	PX567951	PX576710	PX578648
	An_{7-1}	PX557998	PX576689	PX567949	PX576709	PX578645
	An_{7-2}	PX557999	PX576690	-	PX576711	PX578646
	An_{7-3}	PX558000	PX576691	PX567950	PX576702	PX578647
	An_{8}	PX557997	PX576701	PX567948	PX576708	PX578644
	An_{9}	PX557992	PX576697	PX567955	PX576703	PX578653

2.4 Sequence of reference strains

Table S4 Strains and sequences used in molecular study

Species	Strains No.	GenBank accession No.				
		ITS	*BenA*	*LSU*	*CaM*	*RPB2*
Penicillium eremophilus	CBS123361	NR160230.1	KY709170.1	AF365023.1	KY611931.1	KY611970.1
Monascus recifensis	URM 7523	KY511739	KY709156	KY511769	KY611917	KY611956
M. recifensis	URM 7524	KY511740	KY709157	KY511770	KY611918	KY611957
Monascus flavipigmentosus	URM 7534	KY511750	KY709167	KY511780	KY611928	KY611967
M. flavipigmentosus	URM 7535	KY511752	KY709169	KY511782	KY611930	KY611969
M. flavipigmentosus	URM 7536	KY511751	KY709168	KY511781	KY611929	KY611968
Monascus mellicola	URM 7522	KY511738	KY709155	KY511768	KY611916	KY611955
M. mellicola	URM 7520	KY511736	KY709153	KY511766	KY611914	KY611953
M. mellicola	URM 7507	KY511723	KY709140	KY511753	KY611901	KY611940
M. mellicola	URM 7514	KY511730	KY709147	KY511760	KY611908	KY611947
M. mellicola	URM 7521	KY511737	KY709154	KY511767	KY611915	KY611954
M. mellicola	URM 7510	KY511726	KY709143	KY511756	KY611904	KY611943
Monascus ruber	URM 7531	KY511747	KY709164	KY511777	KY611925	KY611964
M. ruber	URM 7527	KY511743	KY709160	KY511773	KY611921	KY611960
M. ruber	URM 7529	KY511745	KY709162	KY511775	KY611923	KY611962
M. ruber	URM 7525	KY511741	KY709158	KY511771	KY611919	KY611958
M. ruber	URM 7528	KY511744	KY709161	KY511774	KY611922	KY611961
M. ruber	URM 7533	KY511749	KY709166	KY511779	KY611927	KY611966
M. ruber	URM 7526	KY511742	KY709159	KY511772	KY611920	KY611959
Monascus purpureus	CBS 109.07	KY635851	KY709176	KY635859	KY611937	JN121422
Aspergillus brasiliensis	CMV007D1	MK450631	MK451119	-	MK451325	MK450774
A. brasiliensis	NRRL26651	KC796389	EF661094	-	EF661160	EF661064
A. brasiliensis	ITEM 6139	AM295181	AM295185	-	AM295176	OP081976
A. brasiliensis	IHEM 5185	MH613109	MH614565	-	MH644892	OP081982
A. brasiliensis	CBS 101740	FJ629321	FJ629272	-	FN594543	EF661063
Aspergillus niger	NRRL 326	EF661186	EF661089	-	EF661154	EF661058
A. niger	IHEM18069	MH613155	MH614489	-	MH645000	OP082110

A. niger	IHEM 2312	MH613218	MH614521	-	MH645010	OP082127
A. niger	ITAL 47.456	MN575692	MN583579	-	MN583580	MN583581
A. niger	IHEM22373	KP131600	MH614468	-	MH644935	OP082121
A. niger	IHEM14389	KP131597	MH614517	-	MH644975	OP082106
A. niger	IHEM 3019	MH613206	MH614514	-	MH644970	OP082158
A. niger	IHEM 4461	KP131607	MH614585	-	MH644978	OP082164
Aspergillus tubingensis	NRRL 4875	EF661193	EF661086	-	EF661151	EF661055
A. tubingensis	IHEM18329	MH613202	MH614467	-	MH644992	OP082002
A. tubingensis	IHEM18097	MH613119	MH614417	-	MH644981	OP081998
A. tubingensis	CMV005A5	MK450660	MK451021	-	MK451542	MK450813
A. tubingensis	CBS 134.48	FJ629354	AY820007	-	AJ964876	EF661055
A. tubingensis	CMV001B7	MK450658	MK450891	-	MK451540	MK450811
A. tubingensis	CMV005A3	MK450659	MK451019	-	MK451541	MK450812
A. tubingensis	IHEM22370	KP131632	MH614525	-	MH644894	OP082014
A. tubingensis	CBS 553.65	FJ629327	FJ629278	-	OP081904	OP081983
Aspergillus carbonarius	NRRL 369	EF661204	EF661099	-	EF661167	EF661068
A. carbonarius	NRRL 67	EF661202	EF661097	-	EF661165	EF661066
A. carbonarius	NRRL 4849	EF661205	EF661100	-	EF661168	EF661069
A. carbonarius	NRRL 346	EF661203	EF661098	-	EF661166	EF661067

Other reference sequences of *Monascus* spp. were obtained by pre-sequencing in our laboratory and have not yet been uploaded to NCBI.

3. Morphological observation

3.1 Colonial morphologies results

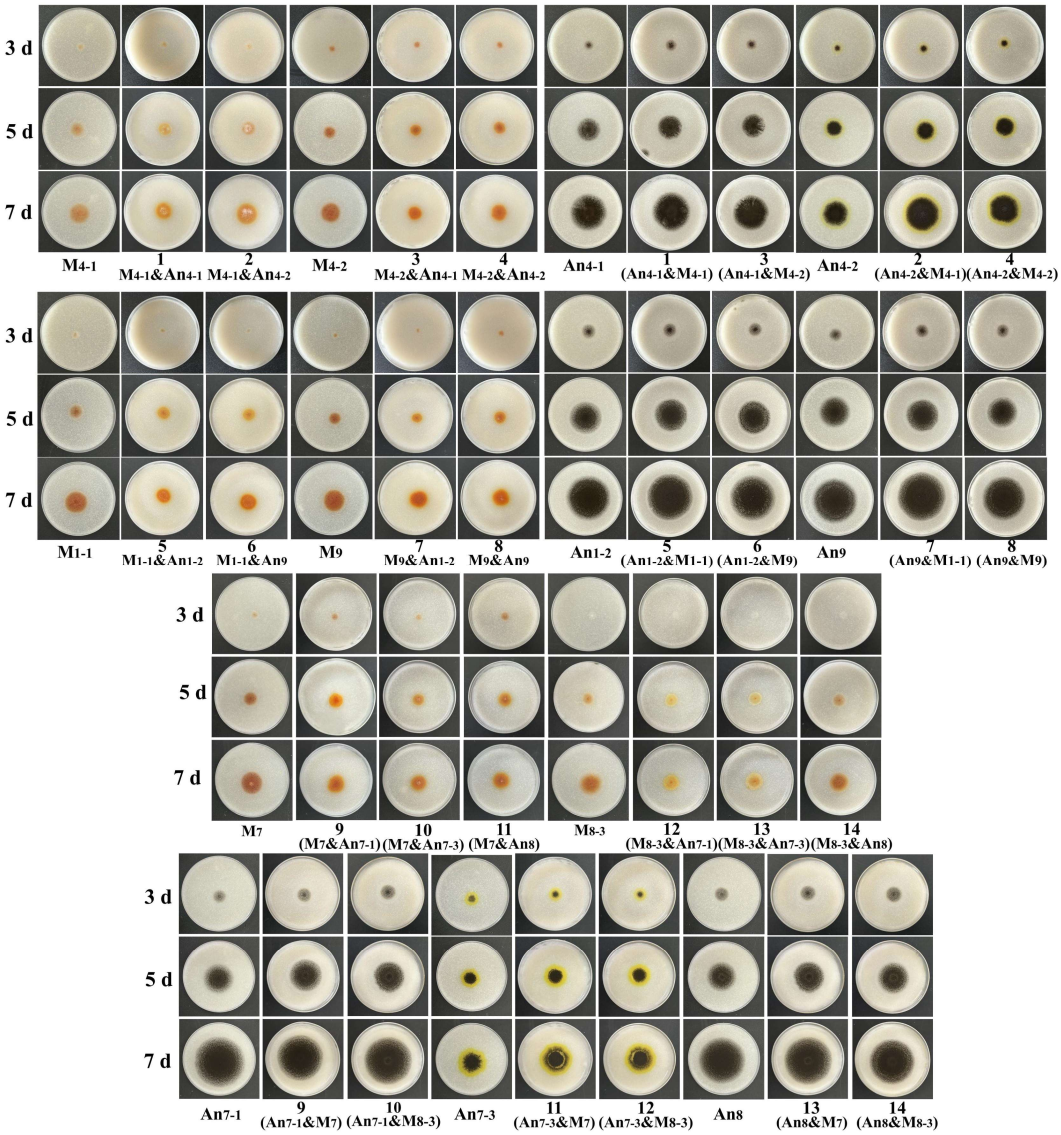

Figure S4 Colonial morphologies of *M. purpureus* and *A. niger* co-cultured using DSPD on RA media at 28°C

3.2 Microscopic morphologies results

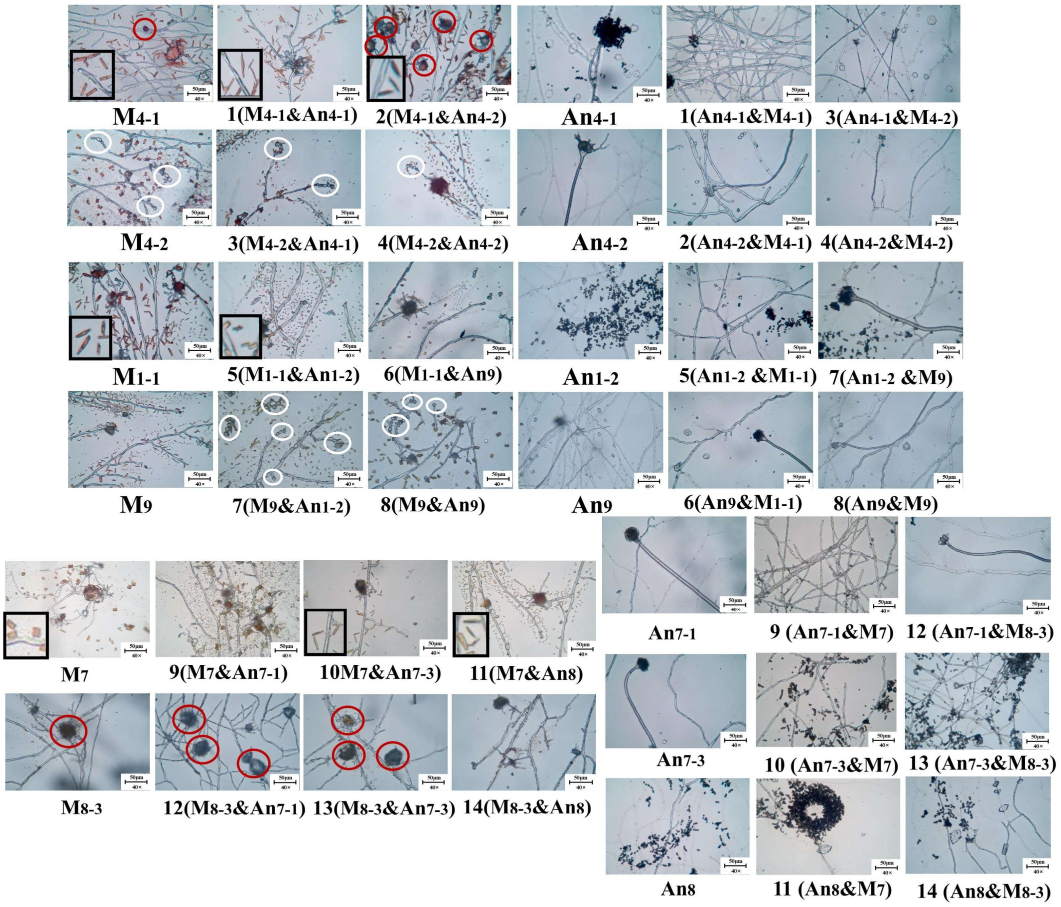

Figure S5 Microscopic morphologies of *M. purpureus* and *A. niger* co-cultured using DSPD on RA media at 28°C

4. High performance liquid chromatography

4.1 Liquid chromatography of MPs

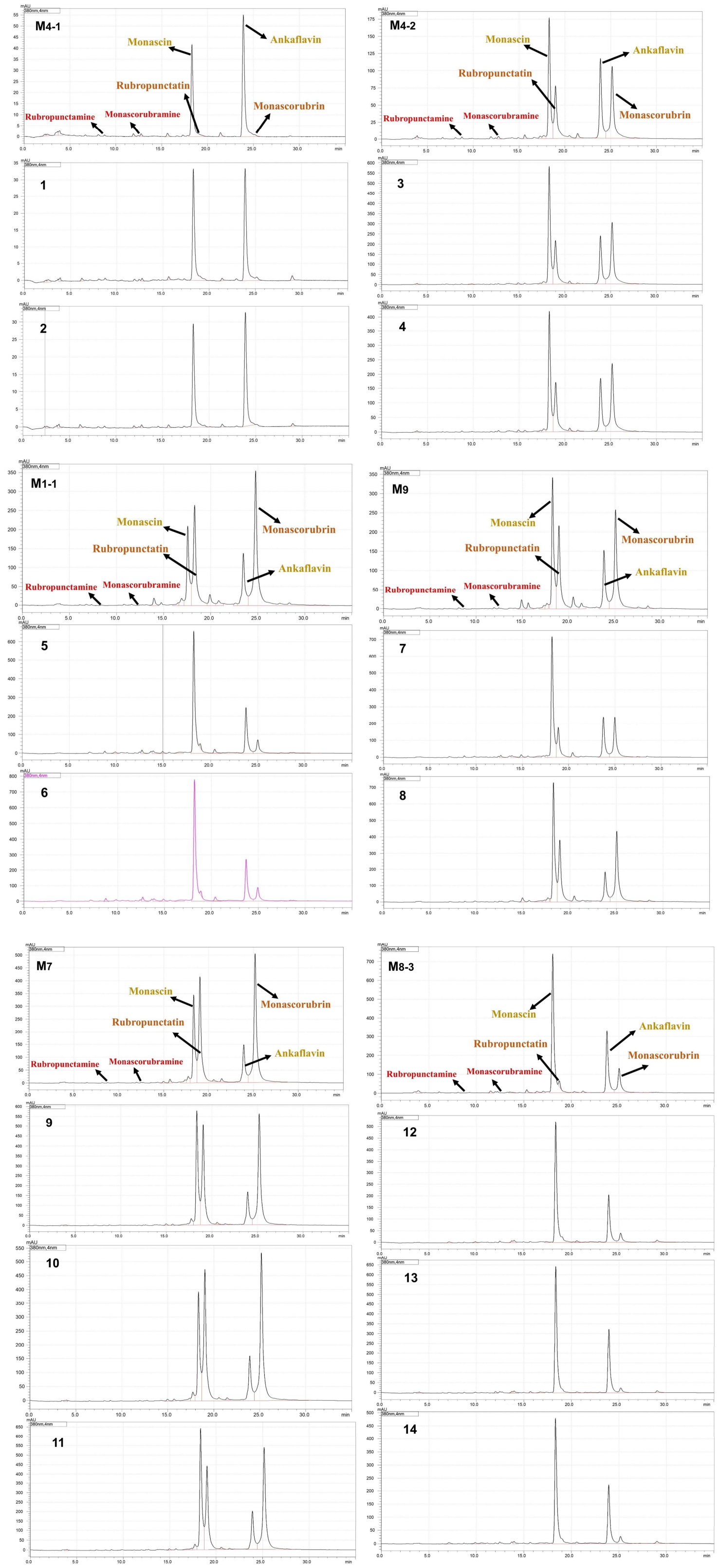

Figure S6 HPLC chromatography diagram of MPs

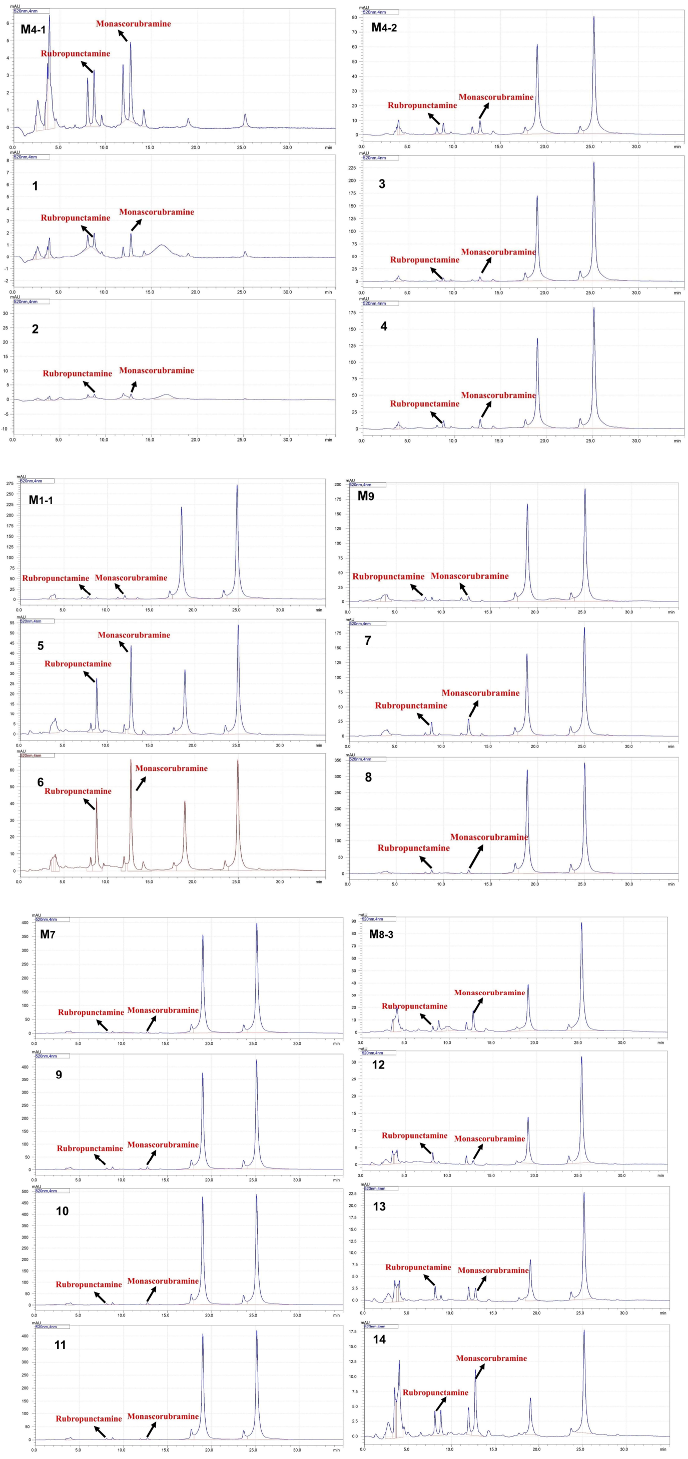

Figure S7 HPLC chromatography diagram of *Monascus* red pigments

4.2 Liquid chromatography of CIT

4.2.1 Intracellular CIT

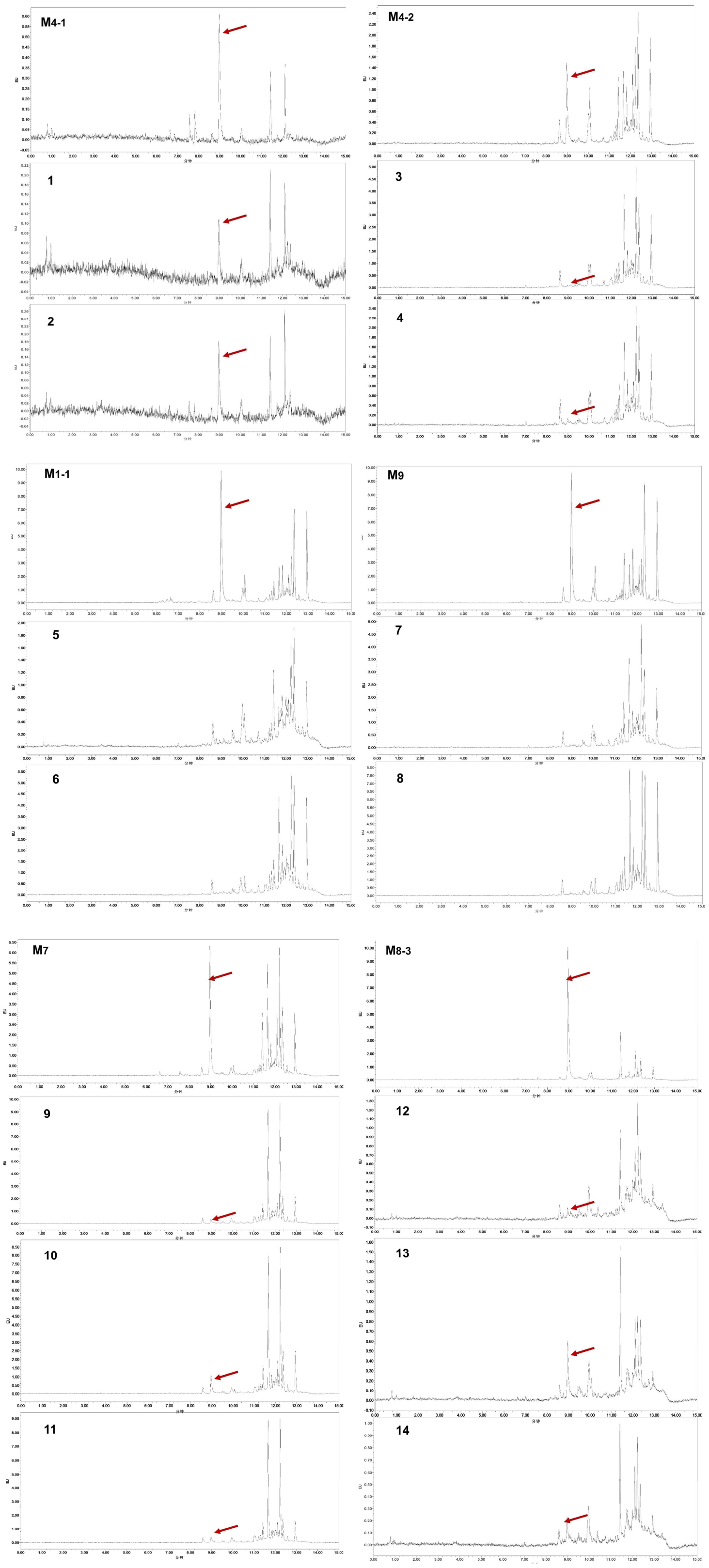

Figure S8 UPLC chromatography diagram of intracellular CIT

4.2.2 Extracellular CIT

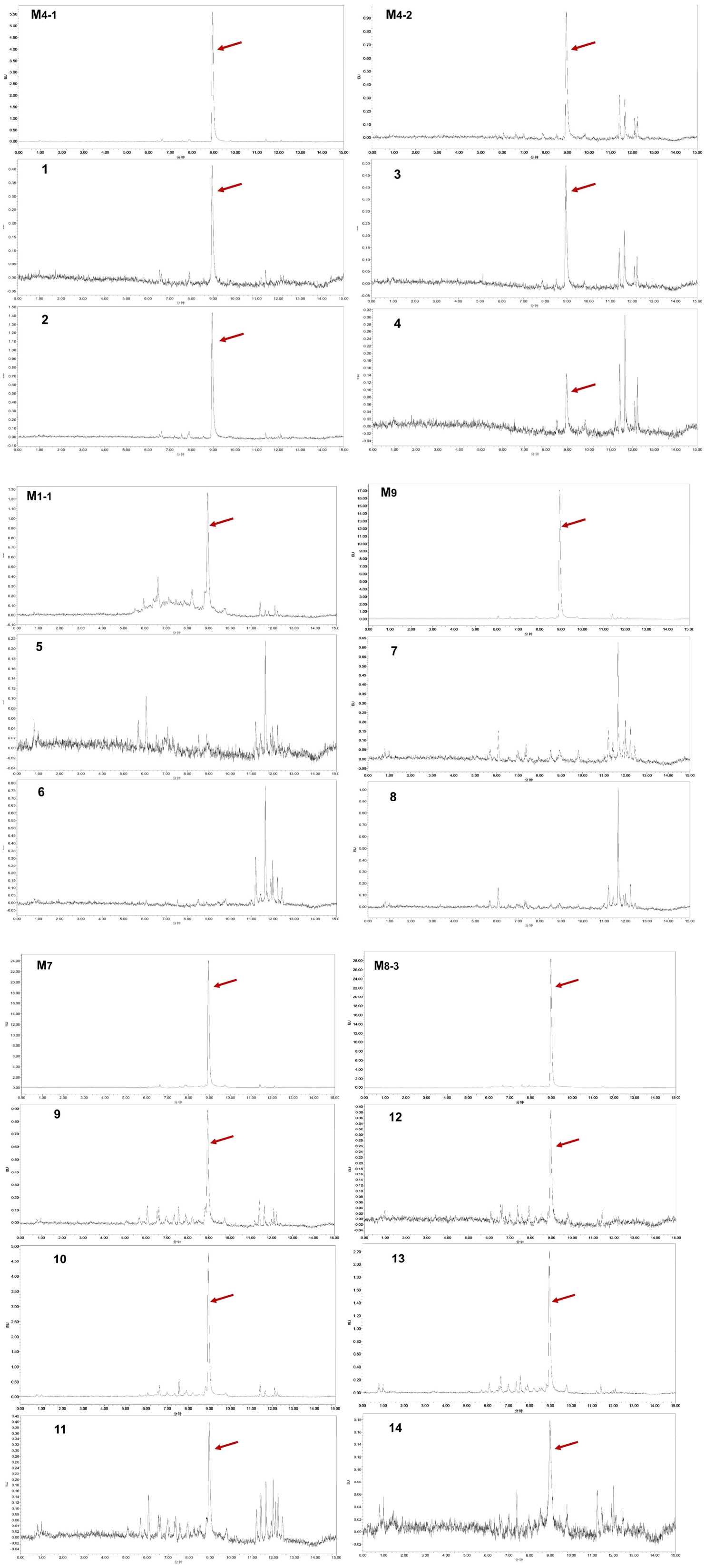

Figure S9 UPLC chromatography diagram of extracellular CIT

5. RT-qPCR

5.1 Electrophoresis of total RNA

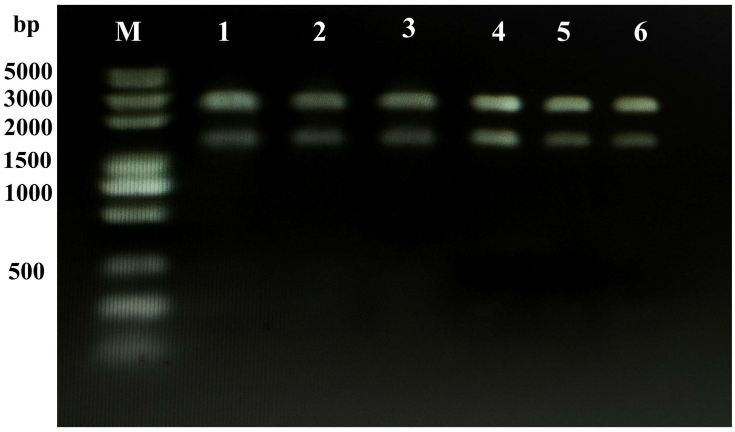

Figure S10 Total RNA of 6 samples for RNA in 1% agarose gel electrophoresis

M: *Trans* 2K plus DNA Maker; Lane 1~3: M_{1-1} (Monoculture), M_{1-1}&An_{1-2}, M_{1-1}&An_{9};

Lane 4~6: M_{9} (Monoculture), M_{9}&An_{1-2}, M_{9}&An_{9}

5.2 RT-qPCR related primers

Table S5 Primers for RT-qPCR of *Monascus* MPs and CIT biosynthetic genes

Gene cluster	Genes	Forward primers (5'→3')	Reverse primers (5'→3')	Primers Concentrations(ng/μL)
Internal reference gene	*β-actin*	TCTGGCACCACACATTCTACAA	CGAAGACGATCTGGGTCATCT	F: 303.1; R: 308.3
MPs gene cluster	*pigA*	CTCGAGGAATTGAGCGTTGG	CAGGAAGACTCAATTCGCCG	F: 324.5; R: 324.5
	pigB	CAGAAACCATCACGCAGGAG	AAAGAAGCAGCGGGTCTACT	F: 301.7; R: 306.2
	pigC	CCTACCCAGCAATCGATCCT	ACGTCCTTTGCTAGCTCTGT	F: 367.2; R: 386.4
	pigD	GTACGCGGGGAAGTTCAATC	CCCCAATATCCTCCCTCGTC	F: 311.9; R: 329.0
	pigE	CTGTACAACGTCCTGCATCG	TCTCCCGAATCGTATCCAGC	F: 362.3; R: 354.8
	pigF	GTCACGTCTCAGATCGCAAC	CTGGCACTGTCGATGAACTG	F: 344.6; R: 337.2
	pigG	TACAAGGAGTTCGGGCCATT	GCAGGCTAGCACACATCTTC	F: 338.4; R: 357.6
	pigH	TCGTCTCGTGGATCATCTCG	GATGCTCTCCAATCCCTTGC	F: 385.2; R: 390.5
	pigI	CATCTTGGACGGGATTGCAG	ATCTCGTCCTTGCTCACACA	F: 383.1; R: 367.9
	pigJ	CGTTTCGGCTGATCATTCGT	CGATCCCGCTGAAGAACTTG	F: 388.6; R: 376.5
	pigK	CAATCGGACGGGAAATGACC	CTTTGAGTCTCATCGCCAGC	F: 357.8; R: 372.3
	pigM	GTGACTTTGAACAGCCTGGG	CGCTCAATTCCTTCTCCAGC	F: 354.5; R: 386.8
	pigN	CGATGCAATGGGGAGAGAGA	CGAATCCAGAGAAGGCTTGC	F: 317.9; R: 322.1
	pigO	AACTGCTCTTCGAGACGGAT	CGAACTCCAGCAGCAACTTC	F: 347.6; R: 355.8
	pigP	CTATTTGGTGCGGACGAGTG	TCCAACACCTCTTCGATGCT	F: 353.0; R: 361.8
CIT gene cluster	*citA*	GTCTGATGCTGAGCCCAATG	CGGATTTGTTGGCGGTAGAG	F: 322.4; R: 338.7
	citB	AGACATTCCCCTCGACGATC	GCGACAAGGTCCAACACTAC	F: 352.1; R: 349.6
	citC	GAAGTGATTCTCAGCGCTGG	AGAGCGGGATCCTTGAACAA	F: 318.3; R: 335.8
	citD	CGCTTGTCAAGATGGTCTCG	TCGCCCTTGTTGAAGAAAGC	F: 376.5; R: 362.9
	citE	GCCATGCTGCCTCTTCTTTT	ACTTTGCCTTGGTGTCTTCG	F: 385.6; R: 374.1
	citS	AAGCCAATATTCAGCGCCTG	GCACCAGTAACAAGCACACA	F: 312.4; R: 308.6
	ctnA	CCGAGCCAGACATGTCATTG	GATGTCTTCTTCGCGGCATT	F: 357.9; R: 362.5

PCR amplification reaction system：

2×qPCR Mix	10 μL
10 μM forward primers	0.4 μL
10 μM reverse primers	0.4 μL
Reverse transcription product cDNA	2.0 μL
ddH_2O	7.2 μL

The PCR amplification procedures were: pre-denaturation at 95°C for 5 min, denaturation at 94°C for 10 s, annealing at 60°C for 30 s, cycling for 40 times.
